# The predictive value of Hounsfield units for titanium mesh cage subsidence after anterior cervical corpectomy and fusion

**DOI:** 10.3389/fsurg.2022.1012364

**Published:** 2023-01-06

**Authors:** Haimiti Abudouaini, Tingkui Wu, Hao Liu, Beiyu Wang, Hua Chen

**Affiliations:** Department of Orthopedic Surgery, West China Hospital, Sichuan University, Chengdu, China

**Keywords:** anterior cervical corpectomy and fusion, ACCF, Hounsfield units, subsidence, bone mineral density

## Abstract

**Objective:**

To investigate whether bone mineral density (BMD) measured in Hounsfield units (HUs) correlates with titanium mesh cage (TMC) subsidence after anterior cervical corpectomy and fusion (ACCF).

**Methods:**

A total of 64 patients who underwent one or two levels of ACCF with TMC with a mean follow-up of 19.34 ± 7.86 months were analysed. HU values were measured three times in 3 different planes in the upper and lower vertebrae according to published methods. Subsidence was defined as segmental height loss of more than 3 mm. Pearson correlation analysis was performed. Receiver operating characteristic (ROC) curve analysis was used to obtain optimal thresholds. A multivariate logistic regression analysis was also conducted.

**Results:**

Twenty-two patients (34.38%) had evidence of TMC subsidence on follow-up x-ray. The mean HU values in the subsidence group (317.34 ± 32.32, *n* = 22) were significantly lower than those in the nonsubsidence group (363.07 ± 25.23 *n* = 42, *p* < 0.001, *t* test). At last follow-up, mean disc height loss was 4.80 ± 1.16 mm in the subsidence group and 1.85 ± 1.14 mm in the nonsubsidence group (*p* < 0.001). There was a negative correlation between HU values and disc height loss (Pearson's coefficient −0.494, *p* < 0.001). HU values decreased gradually from the C3 vertebra to the C7 vertebra, and the HU values of the C5, C6, and C7 vertebrae in the nonsubsidence group were significantly higher than those in the subsidence group (*p* < 0.05). Furthermore, there were significant differences between the groups in the segmental angle at the last follow-up and the mean changes in segmental angle (*p* < 0.05). The area under the ROC curve was 0.859, and the most appropriate threshold of the HU value was 330.5 (sensitivity 100%, specificity 72.7%). The multivariate logistic regression analysis showed that older age (*p* = 0.033, OR = 0.879), lower LIV HU value (*p* < 0.001, OR = 1.053) and a greater segmental angle change (*p* = 0.002, OR 6.442) were significantly associated with a higher incidence of TMC subsidence after ACCF.

**Conclusion:**

There are strong correlations between a lower HU value and TMC subsidence after ACCF. More accurate assessment of bone quality may be obtained if HU measurement can be used as a routine preoperative screening method together with DXA. For patients with HU values <330.5, a more comprehensive and cautious preoperative plan should be implemented to reduce TMC subsidence.

## Introduction

Reconstruction of the cervical spine with titanium mesh cages (TMCs) has been widely used in anterior cervical corpectomy and fusion (ACCF) (1, 2). Although many previous studies report satisfactory decompression, rapid stabilization and a relatively high fusion rate (ranging from 95% to 100%) (3), subsidence of the TMC is a major concern before implementing ACCF. Indeed, cage subsidence after ACCF may lead to ligamentum flavum wrinkles and neural foramen stenosis, segmental instability, non-union, or postoperative kyphosis, with some patients even requiring revision surgery (4, 5).

Several papers have reported a close relationship between bone mineral density (BMD) and postoperative cage subsidence in both the lumbar and cervical spines (6–8). The DXA (dual-energy x-ray absorptiometry) technique is widely used to evaluate preoperative bone quality before spinal surgery. DXA assesses BMD by measuring the T-score of both hips and waist 1–4 to represent the whole-body bone quality. However, previous literature shows that measurement error may occur when evaluating the bone mass of spine vertebra using DXA examination (9–11). Moreover, there is still insufficient evidence to prove the accuracy of assessing the bone mass of the cervical vertebrae with T-score measured in the hip bone and lumbar spine. Although local trabecular BMD can be accurately obtained through quantitative CT (QCT) (12), the popularity of QCT remains very low in China and is also costly.

Several studies have found that Hounsfield units (HU) measured by CT are associated with cage subsidence after lumbar surgery (13–15). To the best of our knowledge, there are few reports on the relationship between TMC subsidence and HU values in ACCF (16). Therefore, in this study, we sought to determine associations between preoperative CT HU and post-ACCF TMC subsidence and to identify patients who are at high risk for severe subsidence.

## Materials and methods

### Study population and criteria

Patients who underwent single- and two-level ACCF using a titanium mesh cage (Medtronic Sofamor Danek) from March 2011 to December 2019 were included. All surgeries were performed by one surgeon. The study's indication for ACCF included posterior osteophytes of the vertebrae, ossified posterior longitudinal ligament (OPLL) and prolapse of the free nucleus pulposus between the C3/4 and C6/7 levels that did not respond to conservative treatment for at least 6 weeks or resulted in progressive symptoms of nerve root/spinal cord compression. All patients were followed up clinically and radiographically for a minimum of 12 months. Patients undergoing anterior cervical discectomy and fusion (ACDF), revision surgery and history of surgery, trauma or tumor at the C1–C7 level, and severe osteoporosis (T-score ≤−2.5), Patients with endplate injury were excluded. Besides, patients with lack of clinical and radiological data or lost follow-up were also excluded and patients only with complete clinical, radiological, and follow-up data were included.

### Surgical procedure

After general anesthesia, the patient was maintained in the supine position with the neck slightly extended. The Smith-Robinson approach was used in all cases. After the intervertebral space was expanded, discectomy and removal of vertebral bodies were performed, and autologous bone was applied as bone graft material. Then, the osteophytes and posterior longitudinal ligament were removed, and the endplate was carefully prepared. After adequate decompression, a TMC with an appropriate size was selected and filled with autologous bone fragments. The TMC was then inserted into the corpectomy defect, and fluoroscopy was used to confirm the cage location. Last, a suitably sized anterior cervical locking plate system was used in all cases for further stabilization. After surgery, all patients were advised to wear a soft neck collar for 6 weeks.

### Radiographic evaluation

**Figure 1:**
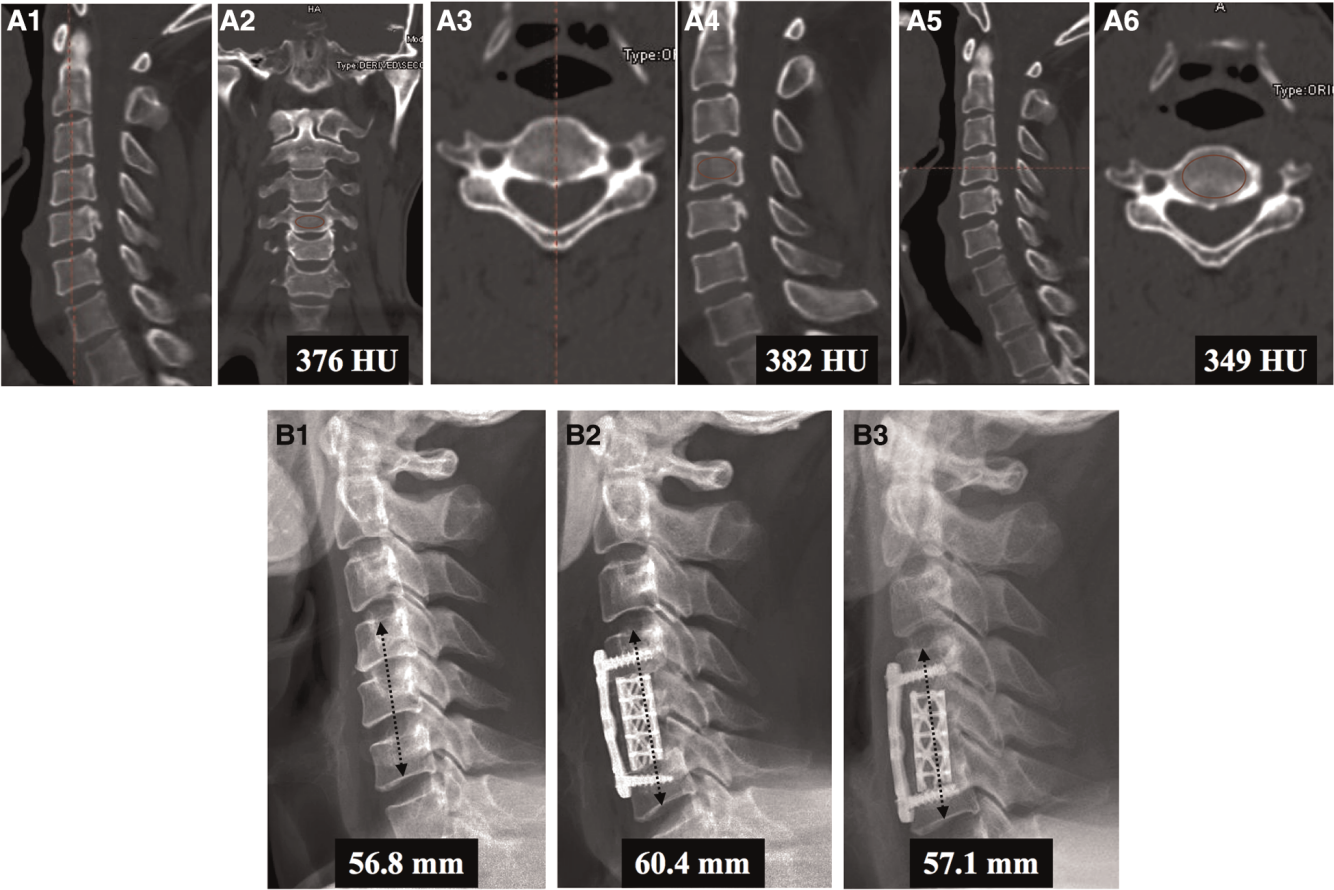
Measurement method of the HU value in C5 ACCF. (**A**) First, HU values of the C4 vertebral body were calculated. The HU values of the mid-sagittal (**A1,A2**), mid-coronal (**A3,A4**), and mid-axial (**A5,A6**) planes were measured. The same method was used to calculate the HU values of the C6 vertebral body. (**B**) Illustration of the method for measuring disc height. Disc height was defined as the straight line between the midpoint of the lower endplate of the upper vertebra and the midpoint of the upper endplate of the lower vertebra. Disc height was measured before surgery (**B1**) and within 3 days after surgery (**B2**) and at the final follow-up (**B3**). This patient was assessed to have subsidence due to height loss greater than 3 mm at the final follow-up (1.5 years after surgery).

**Figure 2:**
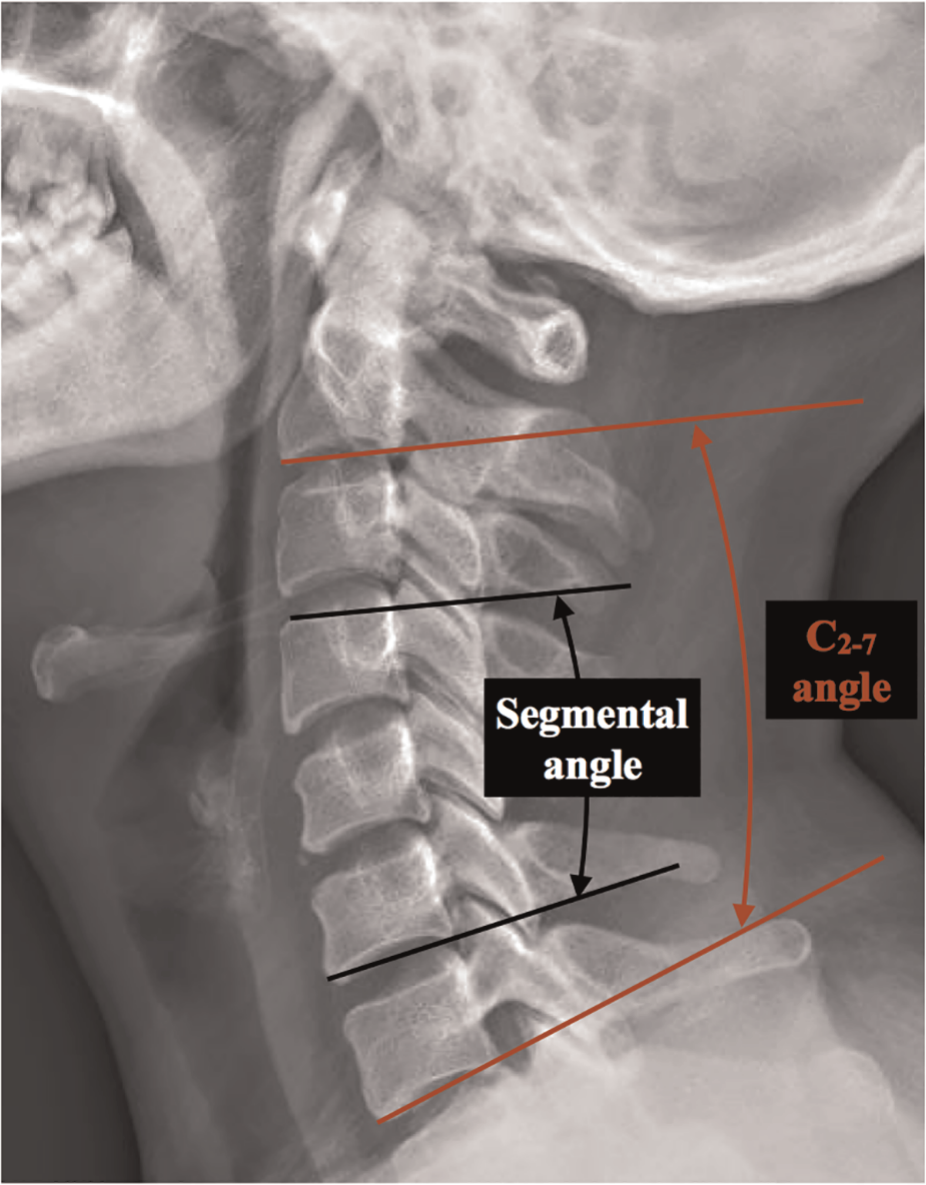
Measurement method of the global and segmental cervical curvature. The C2–7 angle was defined as the angle between the caudal margin of C2 and the caudal margin of C7 in a neutral position. The segmental angle was defined as the angle formed by lines drawn at the cranial margin of the upper instrumented vertebral body and the caudal margin of the lower instrumented vertebral body.

HU was measured at vertebrae above and below the titanium mesh cage placement (e.g., C4 ACCF had C3 and C5 vertebral bodies measured for HU). The measurement method proposed by Schreiber et al. (17) was used to evaluate the vertebral body HU values. The HU value was measured three times in the upper and lower vertebrae by selecting the elliptical region of interest (ROI) on sagittal, mid-coronal and mid-axial plane CT image reconstruction, and the average value was defined as the final HU (Figure 1A). The connecting line between the midpoint of the upper endplate of the upper vertebra and the midpoint of the lower endplate of the lower vertebra was defined as the disc height (Figure 1B). Disc height measurements were recorded before surgery, at the initial postoperative radiograph (within 7 days after surgery) and at the final follow-up; the difference between the last follow-up and initial postoperative disc height was defined as the loss in disc height. Subsidence was defined as a disc height loss >3 mm or TMC migration (angular deviation >3 degrees in lateral and AP plane) into the endplate at the final follow-up (4). The C_2–7_ angle was the angle between the caudal margin of C_2_ and the caudal margin of C_7_ at the neutral position. The segmental angle was the angle formed by lines drawn at the cranial margin of the superior vertebral body and at the caudal margin of the inferior body (Figure 2). Solid bone fusion was defined as the establishment of a solid bone bridge between fusion segments on the last follow up reconstructed CT scans.

To reduce measurement errors, an independent panel of radiologists was established in our study. The panel consisted of three study-blinded radiologists. Among them, two radiologists were responsible for data collection while the remaining one was responsible for data analysis. In case differences between the first two collected sets of data were relatively large (e.g., more than 10 HU value, 2 degrees or 2 mm), the third radiologist was responsible for confirmative remeasuring.

### Statistical methods

All statistical analyses were performed using SPSS 25.0 software (SPSS Inc., Chicago, IL, USA). Continuous data are presented as the mean ± standard deviation. Independent t tests were used to detect differences, including HU values and disc height, between patients with and without TMC subsidence. Chi-square analysis and Fisher's exact test were applied to assess differences between groups for categorical variables. Correlations were analysed using Pearson correlation. The optimal cut-offs of the HU value were established using a receiver operating characteristic (ROC) curve, and the area under the curve (AUC) was calculated. The maximum Youden index was used to determine the optimal cut-off value of Hounsfield units. Patients were classified according to the HU threshold value, as determined by ROC curve analysis. A multivariate logistic regression analysis was also conducted. For all statistical analyses, 95% confidence intervals were obtained; *p* < 0.05 (two-sided) was the criterion for statistical significance.

## Results

A total of 64 patients met the inclusion criteria (Table 1). All patients were followed up for at least 1 year (mean 19.34 ± 7.86 months). Of the 64 patients, 22 (34.38%) developed TMC subsidence, and 42 were classified into the nonsubsidence group. There were no significant differences in sex, BMI, level number or distribution, T-score between the subsidence and nonsubsidence groups (Table 2). The average age in the subsidence group was 55.85 ± 9.43 and 48.95 ± 9.54 years in nonsubsidence group. The average age in subsidence group was significantly higher than the average age in nonsubsidence group (*p* = 0.017). The mean HU value in the subsidence group was 317.34 ± 32.32, which was significantly lower than the mean HU value of the nonsubsidence group, at 363.07 ± 25.23 (*p* < 0.001; Table 2). The mean height loss in the nonsubsidence and subsidence groups was 1.85 ± 1.14 and 4.80 ± 1.16 mm, respectively (*p* < 0.001; Table 2).

**Table 1:** Preoperative information and disc height of the patients.

Variable	Value
Number of patients	64
Age (years)	51.29 ± 9.98
BMI (kg/m^2^)	23.16 ± 3.68
Sex (*n*)	
Male	37
Female	27
Follow-up (months)	19.34 ± 7.86
Level number and distribution (*n*)	
One level	
C4	10
C5	36
C6	13
Two level	
C4 + 5	1
C5 + 6	4
T-score	−0.24 ± 1.30
Disc height (mm)	
Preoperative	49.10 ± 5.26
1 week	64.80 ± 4.32
Final follow-up	59.02 ± 4.83
Mean change	2.86 ± 1.82

Change of disc height, disc height at 1 week after surgery—disc height at last follow-up.

**Table 2:** Preoperative information and HU between patients with and patients without subsidence.

Variable	Subsidence group (*n* = 22)	Control group (*n* = 42)	*p* value
Age (years)	55.85 ± 9.43	48.95 ± 9.54	**0** **.** **017**
BMI (kg/m^2^)	23.02 ± 3.46	23.23 ± 3.82	0.737
Sex (*n*)			0.274
Male	14	23	
Female	8	19	
Follow-up (months)	19.09 ± 6.58	19.48 ± 8.52	0.915
One/Two level	21/1	38/4	0.371
T-score	−0.46 ± 1.21	−0.13 ± 1.34	0.334
Mean HU value	317.34 ± 32.32	363.07 ± 25.23	**<0** **.** **001** ^a^
No. >330.5 HU	6	42	**0** **.** **001** **^b^**
No. <330.5 HU	16	0	
Mean height loss (mm)	4.80 ± 1.16	1.85 ± 1.14	**<0** **.** **001** ^a^

Bold values represents that the results have statistical significance.

^a^ Independent *t*-test.

^b^ Fisher's exact test.

**Figure 3:**
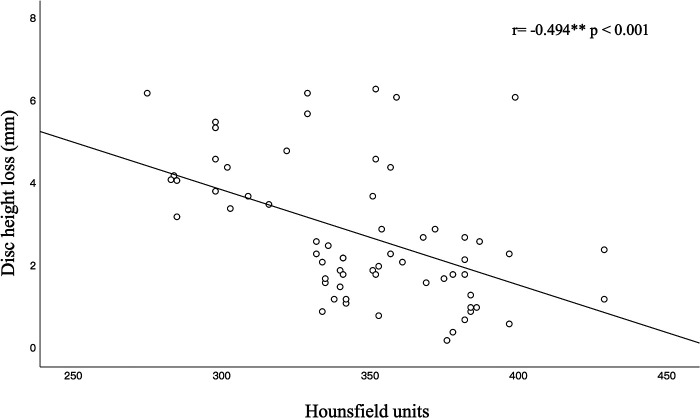
Linear correlation (black line) between HU and the TMC subsidence correlation coefficient (*r*) at the overall cervical vertebrae. Significant correlation coefficients are indicated by asterisks.

Pearson correlation analysis revealed a significant negative correlation between preoperative HU values and postoperative disc height loss (*r* = −0.494, *p* < 0.001; Figure 3). HU values decreased gradually from the C3 vertebra to the C7 vertebra, and the HU values of the C5, C6, and C7 vertebrae in the nonsubsidence group were significantly higher than those in the subsidence group (*p* < 0.05, Table 3). Furthermore, there were no significant differences in global cervical curvature between the 2 groups (*p* > 0.05, Table 4). In the nonsubsidence group, the segmental angle was improved from 1.63 ± 2.01° before surgery to 3.26 ± 2.03° at the last follow-up, with a mean change value of −0.15 ± 0.60°. In the subsidence group, it decreased from 1.67 ± 2.78° before surgery to −1.19 ± 4.10 at the last follow-up, and the mean change value was −3.73 ± 3.53. There were significant differences between the groups in the segmental angle at the last follow-up and the mean changes in segmental angle (*p* < 0.001) (Table 4).

**Table 3:** Mean HU values of different subaxial cervical vertebrae in patients.

Level	Total (*n* = 64)	Mean vertebral HU		*p* value
		Subsidence (*n* = 22)	Non-subsidence (*n* = 42)	
C3	397.18 ± 42.54	398.67 ± 64.08	397.03 ± 41.40	0.951
C4	383.09 ± 57.13	378.52 ± 55.30	385.55 ± 58.30	0.523
C5	354.57 ± 62.15	311.63 ± 52.26	376.04 ± 55.67	**<0** **.** **001**
C6	312.04 ± 54.29	276.12 ± 40.08	332.20 ± 50.47	**<0** **.** **001**
C7	297.10 ± 42.95	272.38 ± 30.23	319.07 ± 40.91	**<0** **.** **001**

Bold values represents that the results have statistical significance.

**Table 4:** Comparison of the global and segmental cervical curvature between the groups.

	Subsidence group (*n* = 22)	Control group (*n* = 42)	*p* value
C_2–7_ A (°)			
Preoperative	13.18 ± 5.01	12.19 ± 7.35	0.334
1 week	14.52 ± 3.71	13.94 ± 6.76	0.471
Last FU	12.57 ± 2.86	13.35 ± 5.60	0.530
dC_2–7_ A	−1.94 ± 2.57	−0.59 ± 6.73	0.185
Segmental A (°)			
Preoperative	1.67 ± 2.78	1.63 ± 2.01	0.824
1 week	2.53 ± 4.05	3.41 ± 2.00	0.296
Last FU	−1.19 ± 4.10	3.26 ± 2.03	**<0** **.** **001**
dSegmental A	−3.73 ± 3.53	−0.15 ± 0.60	**<0** **.** **001**
Fusion rate (%)	90.91% (20/22)	97.62% (41/42)	0.270

Bold values represents that the results have statistical significance.

C_2–7_ A, C_2_–C_7_ angle; Segmental A, Segmental angle; d, last follow-up value—postoperative 1 week value; Independent *t*-test was used to compare the C_2–7_ A and Segmental A between the groups; *Chi-square test* was used to compare the fusion rate between the groups.

**Figure 4:**
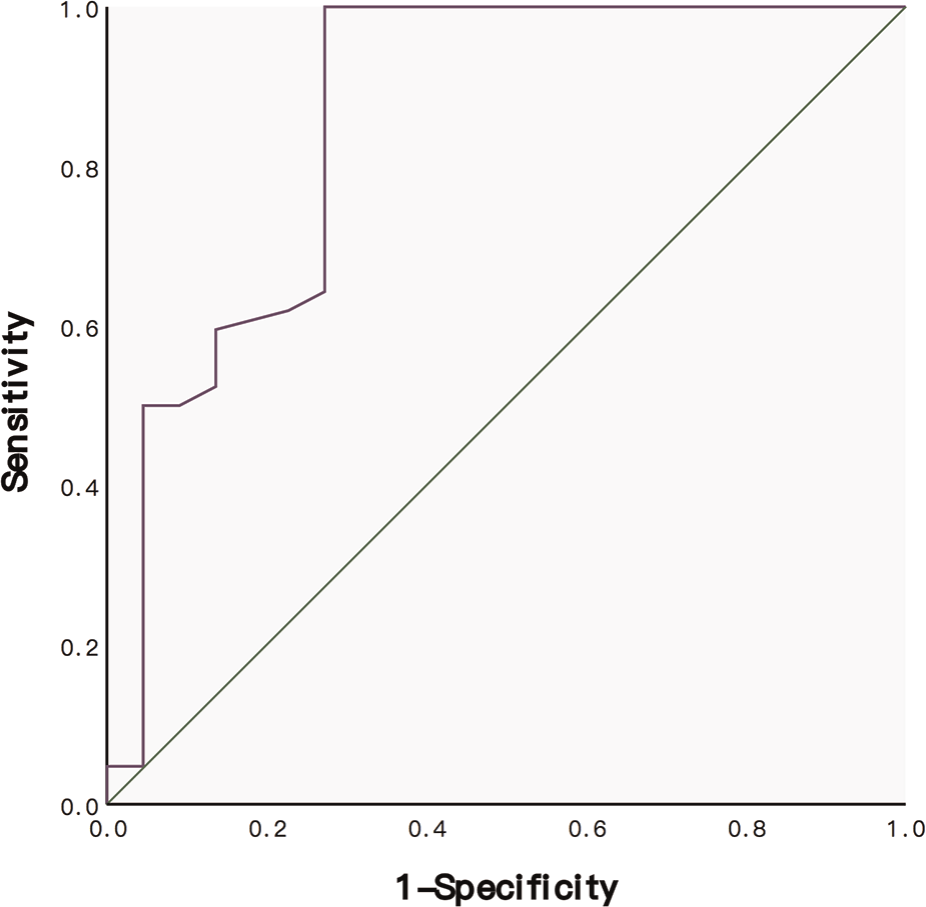
ROC curve for the sensitivity and specificity of HU values in predicting subsidence after ACCF. The AUC was 0.859.

The AUC of the ROC curve was 0.859 (95% CI: 0.748–0.971; Figure 4), and the optimal cut-off was 330.5, with a sensitivity of 100% and a specificity of 72.7%. Fisher's exact test showed that patients with <330.5 HU were more prone towards TMC subsidence (*p* = 0.001, Table 2).

The age, gender, surgical level, HU value of upper instrumented vertebra (UIV) and lower instrumented vertebra (LIV), T-score, segmental angle change and preoperative disc height were elected to undergo multiple logistic regression analysis. The results showed that older age (*p* = 0.033, OR = 0.879), lower LIV HU value (*p* < 0.001, OR = 1.053) and a greater segmental angle change (*p* = 0.002, OR 6.442) were significantly associated with a higher incidence of TMC subsidence after ACCF (Table 5).

**Table 5:** Results of multivariate logistic regression.

Covariates	*B*	*p*	OR	95% CI	
				Lower bound	Upper bound
Age (years)	−0.129	**0** **.** **033**	0.879	0.781	0.989
Gender (male/female)	−0.816	0.507	0.442	0.040	4.937
C6 corpectomy (yes/no)	1.091	0.092	2.978	0.838	10.582
C5 corpectomy (yes/no)	−0.720	0.634	0.487	0.025	9.471
UIV HU value	0.013	0.207	1.014	0.993	1.035
LIV HU value	0.052	**<0** **.** **001**	1.053	1.024	1.082
T-score	0.763	0.135	2.146	0.789	5.834
dSegmental A (°)	1.863	**0** **.** **002**	6.442	1.953	21.253
Preoperative disc height (mm)	−0.224	0.071	0.799	0.627	1.019

Bold values represents that the results have statistical significance.

UIV, upper instrumented vertebra; LIV, lower instrumented vertebra; dSegmental A, change value of segmental angle.

## Discussion

In this study, imaging data of 64 patients undergoing anterior cervical corpectomy with TMC fusion were retrospectively reviewed. TMC subsidence was observed in 22 (34.38%) patients in our study, which is roughly consistent with previous studies (3, 16). However, the subsidence standard varies among studies. Majority of previous articles have used the disc height loss >3mm (4, 16–19) to define the TMC subsidence, whereas others have used 4 mm (20) or 2 mm (21) as a threshold. If subsidence is defined as a definite disc height loss >3 mm, then the subsidence rate of ACCF with TMC fluctuates between 12% and 80% in previous studies (3–5, 17, 22). To the best of our knowledge, there is no consensus in the literature regarding an accurate relationship between TMC subsidence and clinical outcomes. For example, Chen et al. (4) reported that 7% of single-level and 12% of two-level patients with corpectomy developed TMC subsidence, which might have led to poor clinical results and related complications. However, Ji et al. (16) reported that TMC subsidence does not negatively affect the clinical outcomes after ACCF. Nevertheless, nearly all authors agree that low BMD or osteoporosis is an important element causing TMC subsidence. Hence, accurate evaluation of bone quality at the upper and lower adjacent segments seems to be very important to reduce the TMC subsidence rate after ACCF.

In most situations, DXA has been used to assess bone quality prior to performing ACCF. Although DXA is a valid and time-tested method to estimate overall bone quality, it is still unclear whether each cervical vertebra can be accurately assessed. It has been reported that some T-scores obtained by DXA are higher than the actual BMD in patients with severely degenerative spines (9–11). Therefore, HU has received increasing attention during the last few years, with the hope of providing more accurate information on local bone strength. Recent studies have revealed a strong correlation between HU and BMD and T-scores (23–25) and between HUs and graft subsidence (26–28) after spine surgery. However, most of the studies focused on lumbar surgery. To our knowledge, only one article has reported the relationship between the HU value and TMC subsidence after ACCF (16). In that study, the global cervical HU value was significantly lower in the subsidence group (315 ± 73) than in the nonsubsidence group (388 ± 64), and a global cervical HU value <333 was independently associated with TMC subsidence. Nonetheless, the HU value was measured only in axial images: just inferior to the superior endplate, mid-body, and just superior to the inferior endplate. To maximally reduce bias, we measured the HU value 3 times in three different plane; we found that a cut-off value of 330.5 was associated with subsidence and that the HU value decreased gradually from C3–C7. Wang et al. (13) also measured HU values of different subaxial cervical vertebrae before ACDF using the technique described by Schreiber et al. (the same technique was used in our study), and the HU values ranged from 326.9 ± 40.7 to 426.3 ± 61.8 in their study. They also found that a threshold of 343.7 for the HU value had a sensitivity of 77.1% and specificity of 87.5% for predicting cage subsidence after ACDF. In our study, the threshold of 330.5 had a sensitivity of 100% and specificity of 72.7% in predicting cage subsidence for predicting TMC subsidence after ACCF.

Although HU measurement had useful predictive value for both the lumbar and cervical spines, one important limitation of HU measurement in assessing bone quality is that it does not consider various endplate conditions. In fact, several studies have reported that changes in the endplate are associated with cage subsidence (29, 30), and others have reported that CT can evaluate and predict lumbar vertebral endplate mechanical properties, including osteoporosis and osteopaenia (31–33). Therefore, we believe that if appropriate methods that provide more information about the strength of the endplate area in direct contact with the TMC can be added to HU measurements, the predictive value of HU may be further increased.

Lee et al. (8) also analyzed the cervical alignment and segmental angle after ACCF and found that patients with cage subsidence seemed to have significantly greater kyphotic changes than those in patients without subsidence. One different finding from our study, the both cervical alignment and segmental angle were significantly different in their study. However, in our study, a significant difference was only found in segmental angle between the groups. One possible reason is a decrease in segmental height loss during subsidence induces kyphotic changes in the segmental angle, but such changes failed to impact the global cervical curve.

Many factors affecting TMC subsidence after ACCF have been reported in the previous studies. These factors including an older patient age, more corpectomy levels, severe osteoporosis, excessive endplate removal and intraoperative over distraction (20, 29). In addition, the diameter, metal attributes of TMC and the shape of the graft also are important factors that can affect the TMC subsidence after ACCF (34). At present, researchers constantly exploring ways to prevent the subsidence of TMC from many aspects, such as the design of titanium mesh, the exploration of new materials, and surgical methods. Ren et al. (35) have found that middle part of the endplate is mostly a cystic cavity, and the edge of the endplate has the maximum strength. Therefore, endplate should not be scraping too much during the operation, and preserving the integrity of the endplate as possible for preventing postoperative TMC subsidence. Besides, due to cervical spine endplate has a certain inclination, if the TMC cannot be fully contacted to the endplate may lead to stress concentrations and TMC subsidence. Therefore, in order to increase stress dispersion, when inserting TMC during the operation, we should note that both ends of the TMC are as consistent as possible with the inclination of the surface of the endplate. Hasegawa et al. (36) have reported that a TMC with larger diameter and/or augmentation of internal end ring produces a significant increase of the interface strength between the cage and the vertebra, and their result also implies that in severe osteoporotic spine the stability of the cage is declined, and suggested that other instrumentation should be combined with TMC in severe osteoporosis. Based on your suggestion, these contents have been added to the discussion section in the revised manuscript.

## Study limitations

There are some limitations of this study that should be considered. First, this was a retrospective study, and a prospective study is required to confirm the sensitivity and specificity of <330.5 HU in predicting TMC subsidence after ACCF. Second, we did not investigate clinical efficacy because many larger-sample studies have reported the relationship between TMC subsidence and clinical efficacy, which we think may be more representative (4, 16, 5, 17, 22). We did not measured the hardness of endplate in this study. One major reason is that there is currently no standardized methods of evaluate the endplate bone quality directly through Hounsfield units measured on CT. Another limitation of the study is that radiological parameters such as the position and contact area of the TMC were not discussed. This is mainly because we sought to explore the relationship between the bone quality measured by HU values and TMC subsidence, which have some differences from studies that discuss several risk factors at the same time. Besides, even though we measured these radiological parameters based on previous reports, we acknowledge that measurement error may still persists. Another limitation is the relatively small number of patients. Although we selected patients from March 2011 to December 2019, we limited the sample to only operations performed by the same doctor, and ACCF with TMC is not the type of surgery most performed by our team. Similar patients may have more opportunities to undergo multilevel ACDF, cervical disc replacement (CDR) or hybrid surgery (ACDF + CDR) by our team.

## Conclusion

There are strong correlations between a lower HU value and TMC subsidence after ACCF. More accurate assessment of bone quality may be obtained if HU measurement can be used as a routine preoperative screening method together with DXA. For patients with HU values <330.5, a more comprehensive and cautious preoperative plan should be implemented to reduce TMC subsidence.

## Data Availability

The raw data supporting the conclusions of this article will be made available by the authors, without undue reservation.
